# Adipose tissue from oesophageal adenocarcinoma patients is differentially affected by chemotherapy and chemoradiotherapy regimens altering immune cell phenotype and cancer cell metabolism

**DOI:** 10.1016/j.tranon.2025.102302

**Published:** 2025-02-17

**Authors:** Fiona O'Connell, Eimear Mylod, Noel E. Donlon, Maria Davern, Christine Butler, Niamh O'Connor, Meghana S. Menon, Claire L. Donohoe, Narayanasamy Ravi, Derek G. Doherty, Margaret R. Dunne, John V. Reynolds, Helen M. Roche, Jacintha O'Sullivan

**Affiliations:** a Department of Surgery, Trinity St. James's Cancer Institute and Trinity Translational Medicine Institute, St. James's Hospital and Trinity College Dublin, D08 W9RT Dublin, Ireland; b Cancer Immunology and Immunotherapy Group, Department of Surgery, Trinity College Dublin, St. James's Hospital, D08 W9RT Dublin, Ireland; c Department of Immunology, School of Medicine, Trinity College Dublin, Trinity Translational Medicine Institute, St. James's Hospital, Dublin, Ireland; d Nutrigenomics Research Group, UCD Conway Institute, School of Public Health, Physiotherapy and Sports Science, University College Dublin, D04 C1P1 Dublin, Ireland; e Institute for Global Food Security, School of Biological Sciences, Queens University Belfast, Belfast BT9 5DL, UK

**Keywords:** Oesophageal adenocarcinoma, Adipose tissue metabolism, Chemotherapy, Chemoradiotherapy, Myeloid immunology

## Abstract

Oesophageal adenocarcinoma (OAC) is a poor prognosis cancer with limited responses to standard of care treatments including chemotherapy and chemoradiotherapy. OAC has one of the strongest associations with obesity, its anatomical location surrounded by visceral adipose tissue has been postulated to intensify this association. Adipose tissue is a regulatory organ with many unknown downstream functions, including its direct response to chemotherapy and radiotherapy. To elucidate the role of visceral adipose tissue in this disease state, metabolic and secreted pro-inflammatory cytokines analysis was conducted on human *ex-vivo* adipose tissue explants following exposure to FLOT-chemotherapy and CROSS-chemoradiotherapy. To assess how these complex treated microenvironments impact cancer cell metabolism, dendritic cell, and macrophage phenotype, mitochondrial bioenergetics and surface markers expression were examined using seahorse technology and flow cytometry respectively. This study observed that chemotherapy and chemoradiotherapy differentially alter adipose tissue metabolism and secretome, with chemoradiotherapy increasing pro-inflammatory associated mediators (*p<0.05*). The chemoradiotherapy-treated adipose secretome increased cancer cell spare respiratory capacity and dendritic cell adhesion markers (*p<0.05*). In contrast, the chemotherapy-treated adipose microenvironment enhanced mitochondrial dysfunction in cancer cells, increasing their reliance on glycolysis and enhancing pro-inflammatory marker expression on LPS-primed macrophages (*p<0.05*). This study for the first time demonstrates how adipose tissue, and its microenvironment can be significantly impacted by chemotherapy and chemoradiotherapy. These alterations in the adipose secretome in response to therapeutic regimens elicited distinct effects on immune cell phenotype and cancer cells metabolism, raising the question, does the wider tumour microenvironment including the adipose milieu mitigate the efficacy of current treatments.

## Highlights

• FLOT chemotherapy regimen decreased adipose tissue explants from oesophageal cancer patient's utilisation of oxidative phosphorylation and increased glycolytic preference. CROSS chemoradiotherapy regimen increased adipose tissue explants glycolytic preference.
• FLOT chemotherapy regimen decreased adipose tissue explants secretion of pro-inflammatory mediators. CROSS chemoradiotherapy regimen increased adipose tissue explants secretion of pro-inflammatory mediators.
• The adipose secretome treated with FLOT chemotherapy regimen increased oesophageal cancer cell proton leak and utilisation of glycolysis preference. The adipose secretome treated with CROSS chemoradiotherapy regimen increased oesophageal cancer cell spare respiratory capacity and utilisation of oxidative phosphorylation.
• The adipose secretome treated with FLOT chemotherapy regimen increased dendritic cell expression of maturation markers. The adipose secretome treated with either regimen increased DC expression of immunoinhibitory marker TIM-3.
• The adipose secretome treated with FLOT chemotherapy regimen increased unpolarised Mɸ expression of anti-inflammatory associated markers as well as increasing M1-primed Mɸ expression of pro-inflammatory markers and TIM-3. The adipose secretome treated with CROSS chemoradiotherapy regimen increased unpolarised Mɸ expression of pro-inflammatory markers as well as increasing M2-primed Mɸ expression of anti-inflammatory markers.

## Introduction

Chemotherapy and chemoradiotherapy are two of the most historic and commonly used therapies used to treat cancer [1,2]. Currently the standard of care for oesophageal adenocarcinoma (OAC) involves neoadjuvant treatment with either chemotherapy alone, including FLOT regimen, or combination chemoradiotherapy, including CROSS regimen, for locally advanced tumours [3]. Adipose tissue is a regulatory organ with many downstream functions that are not fully understood, including its direct response to chemotherapy and radiotherapy. However, increased visceral adiposity has been reported to have deleterious effects on the efficacy of current cancer treatments including chemotherapy and chemoradiotherapy [4]. Particularly, the efficacy of chemotherapy has been reported to be lessened by adipose tissue, with adipocytes sequestering and metabolising these drugs, diminishing their cytotoxic effects [5]. Chemotherapy has also been reported to diminish lipid storage as well as depleting expression of proteins associated with ATP generation and fatty acid oxidation [6], both key pathways associated with adipocyte metabolism.

The adipose secretome has also been implicated as a key influence on the tumour microenvironment, particularly visceral adipose tissue due to its anatomical distribution and proximity to organs known to develop obesity associated carcinogenesis [7,8]. Chemotherapy and chemoradiotherapy have been reported to augment the secretion of factors from adipose tissue in such a manner that aids cancer metastasis and survival from cytotoxic interventions [9]. The adipose secretome has even been postulated as a potential attractive therapeutic target to potentiate tumour progression and enhance treatment efficacy [8]. Cancer associated adipocytes (CAAs) are one factor that is being actively targeted to improve treatment responses. Targeting of CD36 and its associated mechanisms is currently being explored to mitigate tumour cells lipid uptake from CAAs. CD36 has been linked to promoting chemoresistance in cancer cells and inhibition of CD36 has been reported to evoke immunostimulatory effects and decreased tumour aggressiveness *in-vitro* [10,11]*.* Recent reports have also linked treatment resistance in tumour cells with their ability to invoke a metabolic switch from glycolysis to lipid metabolism to evade the cytotoxic effects of chemotherapy. Inhibition of Fatty acid synthase (FASN) in combination with chemotherapy has been reported to increase the efficacy of these chemotherapy in treatment resistant cancers both *in-vitro* and *in-vivo* [12,13]. This highlights the influential role adipose, and its secreted factors may play in potentiating the efficacy of current cancer treatment modalities.

Mitochondrial dysfunction is commonly induced by cancer cells to aid tumour progression [14]. Aberrant cellular metabolism has been widely reported to be induced by cancer in order to evade the cytotoxic effects of chemotherapy and chemoradiotherapy [15,16]. Particularly, lipid metabolism and fatty acid oxidation has recently been reported to enhance cancer cell resistance to current cancer treatments [17]. Adipose tissue has also been reported to recruit immune cells whilst having deleterious effects on their function which can potentiate anti-tumour immunity [[18], [19], [20], [21], [22]]. Dendritic cells (DC) play a critical role in initiating anti-tumour immunity through antigen presentation. Chemotherapy has previously been suggested to increase DC antigen presentation [23], however induction of DC response following radiotherapy was shown to be dependent on an active immune infiltrate already being present [24].Macrophages, in both their pro-inflammatory and anti-inflammatory phenotypes, play a central role in cancer progression and treatment resistance. Anti-inflammatory or tumour associated macrophages (TAMs) have been reported to be induced by chemotherapy [25] as well as mediating chemoresistance [26]. However, radiation therapy through its initiation of radiation induced inflammation has been linked to the promotion of classically activated macrophages and increased expression of pro-inflammatory mediators which enhance anti-tumour immunity [[27], [28], [29]].

For the first time, this reports on the effects of chemotherapy and chemoradiotherapy on adipose tissue metabolism and its secretome, as well as elucidating the influence of these treated adipose tissue microenvironments on cancer cell metabolism, dendritic cell maturation and macrophage polarisation. This novel data provides information on whether adipose tissue is the mitigating factor in the obesity-cancer link that deleteriously effects the efficacy of current therapies.

## Materials and methods

### Ethics statement and patient recruitment

Ethical approval was granted by the St James's Hospital/AMNCH ethical review board (Ethics number: REC_2019–07 List 25(27)) and written informed consent was collected from all patients in this study. 6 cancer patients were recruited within the period between 8th of January 2022 and 16th of September 2022, patient demographics are listed in Table 1. Fresh adipose tissues were taken from all patients at the start of surgical resection. All OAC patients were being treated with curative intent and had received no treatment prior to surgical resection.

**Figure:**
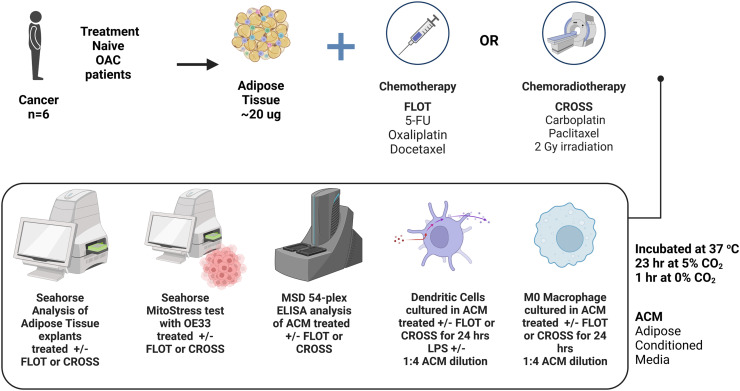
Schematic of experimental methodology workflow Real time metabolic and secreted profiles of adipose explants from OAC treatment naïve cancer patients were assessed by Agilent seahorse and MSD multiplex ELISA following culture with chemotherapy FLOT regimen or chemoradiotherapy CROSS regimen. The influence of these treated adipose secretome on cancer cell metabolism, dendritic cell maturation and macrophage polarisation were then assessed via Agilent Seahorse technology or flow cytometry. Created with BioRender.com.

**Table 1:** Clinical demographics associated with OAC patients.

Patient Clinical Parameters	
**Diagnosis**	OAC = 6
**Sex**	Male = 5 Female = 1
**Age at diagnosis**	53–88 (Mean = 72.5)
**Post-treatment BMI**	25.7–32 (Mean = 29.9)
**Weight**	63–97.2 kg (Mean = 82.15)
**Treatment**	Naïve *n* = 6
**Clinical Stage (T)**	T1 *n* = 4 T3 *n* = 2
**Clinical Stage (N)**	N0 *n* = 5 N1 *n* = 0 N2 *n* = 1
**Path stage (T)**	T0 *n* = 0 T1 *n* = 4 T2 *n* = 2 T3 *n* = 0 T4 *n* = 0
**Path Stage (N)**	N0 *n* = 4 N1 *n* = 1 N2 *n* = 1 N3 *n* = 0

### Seahorse analysis of metabolic profiles from adipose tissue explants and generation of adipose conditioned media (ACM)

Fresh omental tissue was collected from theatre and processed within 30 mins by dissecting into pieces weighing approximately 20 mg. Tissue was plated in triplicates in 1 ml of M199 (supplemented with 0.1 % gentamicin (Lonza, Switzerland), in a 24 well plate (Sarstedt, Germany). Adipose explants were treated with vehicle, FLOT combination chemotherapy (0.08249 μM 5-fluorouracil, 2 μM oxaliplatin, 0.001 μM docetaxel) or CROSS combination chemoradiotherapy (0.001 μM paclitaxel, 1000 μM carboplatin + 2 Gy irradiation). These combination doses were previously optimised in downstream target OE33 cells to achieve 50 % cell death [30]. Following a 2-hour incubation, CROSS-treated adipose explants were irradiated with 2 Gy at a dose rate of 1.73Gy/minute (XStrahl (RS225), Atlanta, United States). Adipose explants were cultured for 24 h at 37 °C at 5 % CO_2_ in a humidified incubator (Thermofisher, Massachusetts, USA). In the last hour of culture, adipose tissue and adipose conditioned media (ACM) was transferred to islet capture microplate with capture screens (Agilent Technologies, California, United States) and incubated in a non-CO_2_ incubator at 37 °C (Whitley, United Kingdom) prior to analysis. Seahorse Xfe24 analyser was used to assess metabolic profiles in adipose explants, (Agilent Technologies, California, United States). Following a 12 min equilibrate step, three basal measurements of OCR (oxygen consumption rate) and ECAR (extracellular acidification rate) were taken over 24 mins consisting of three repeats of the following sequence; mix (3 min) / wait (2 min) / measurement (3 min) to establish basal respiration. ACM was extracted in a sterile environment and tissue weighed using a benchtop analytical balance (Radwag, Poland) and snap frozen. All samples were then stored at −80 °C for further processing.

### Multiplex ELISA

The collected ACM was processed according to MSD (Meso Scale Discovery, Rockville, Maryland, USA) multiplex protocol. To assess angiogenic, vascular injury, pro-inflammatory, and cytokine and chemokine secretions from ACM, a 54-plex ELISA kit separated across seven plates was used (Meso Scale Discovery, Rockville, Maryland, USA). The multiplex kit was used to quantify the secretions of CRP, Eotaxin, Eotaxin-3, FGF(basic), Flt-1, GM-CSF, ICAM-1, IFN-γ, IL-10, IL-12/IL-23p40, IL-12p70, IL-13, IL-15, IL-16, IL-17A, IL-17A/F, IL-17B, IL-17C, IL-17D, IL-1RA, IL-1α, IL-1β, IL-2, IL-21, IL-22, IL-23, IL-27, IL-3, IL-31, IL-4, IL-5, IL-6, IL-7, IL-8, IL-8 (HA), IL-9, IP-10, MCP-1, MCP-4, MDC, MIP-1α, MIP-1β, MIP-3α, PlGF, SAA, TARC, Tie-2, TNF-α, TNF-β, TSLP, VCAM-1, VEGF-A, VEGF-C, and VEGF-D from ACM. All assays were run as per the manufacturer's recommendation, and an overnight supernatant incubation protocol was used for all assays except Angiogenesis Panel 1 and Vascular Injury Panel 2, which were run according to the same-day protocol. ACM was run undiluted for all assays except Vascular Injury Panel 2, where a one-in-four dilution was used, as per previous optimization experiments. Assays were run on a MESO QuickPlex SQ 120, and all analyte concentrations were calculated using Discovery Workbench software (version 4.0). Secretion data for all factors were normalized to adipose post-incubation weight and expressed as pg/mL per gram of adipose tissue.

### Analysis of mitochondrial function of oesophageal cancer cells using Seahorse MitoStress test

The human OE33 oesophageal adenocarcinoma cell line was obtained from the European collection of cell cultures (ECACC, United Kingdom). OE33 cells were seeded at a density of 20,000 in 24 well XFe24 cell culture microplate plate (Agilent Technologies, California, United States) in a volume of 100 μl DMEM (Gibco, Massachusetts, United States) supplemented with 10 % foetal bovine serum (Gibco, Massachusetts, United States), and 1 % penicillin-streptomycin (Lonza, Switzerland). Cells were then allowed to adhere for 3 h before an additional 150 μl of media was added. Cells were rested overnight (approx. 18 h). Media was extracted and replaced with 100 μl M199 control or appropriate treated ACM sample in technical replicates and allowed to incubate for a further 24 h. Following this, cells were washed with Agilent Seahorse XF DMEM Medium supplemented with 10 mM glucose, 1 mM sodium pyruvate, and 2 mM l-glutamine (Agilent Technologies, California, United States) and incubated for 1 hour in a non-CO_2_ incubator at 37 °C. OCR, ECAR, basal respiration, ATP production, maximal respiration, proton leak and non-mitochondrial respiration were assessed using a Seahorse Biosciences XFe24 Extracellular Flux Analyser (Agilent Technologies, California, United States). Three basal measurements of OCR and ECAR were taken over 24 mins consisting of three repeats of mix (3 min) / wait (2 min) / measurement (3 min) to establish basal respiration. Three additional measurements in the same manner were obtained following the injection of 50 μl of 3 mitochondrial inhibitors including 1.8 μM oligomycin (Sigma Aldrich, California, United States), 4 μM Carbonyl cyanide 4- (trifluoromethoxy) phenylhydrazone (FCCP) (Sigma Aldrich, California, United States) and 2 μM Antimycin-A (Sigma Aldrich, California, United States). All inhibitors were diluted in Agilent DMEM. Crystal violet staining was used to assess viability of cells following these cultures and readings were also normalised to this read out. Following assay completion, media was removed, and cells were fixed using 1 % Glutaraldehyde (Sigma Aldrich, California, United States). Cells were washed with PBS and stained with 0.1 % Crystal Violet (Sigma Aldrich, California, United States). Cells were then washed with distilled water and left to airdry overnight. Cells were then incubated with 1 % Triton-X (Sigma Aldrich, California, United States) and shaken at 400 rpm for 1 hour. The resulting supernatant was then transferred to a 96 well plate and absorbance was read at 595 nm on a GloMax Explorer plate reader (Promega, Wisconsin, United States).

### Isolation of monocytes

Peripheral blood mononuclear cells (PBMCs) were obtained from buffy coats (National Blood Centre, St. James's Hospital, Dublin, Ireland). Buffy coats were supplemented with 1 ml of 0.5 mM EDTA and diluted 1:4 with PBS and separated by density gradient centrifugation (Lymphoprep, Thermo Fischer Scientific, Massachusetts, United States) at 400x*g* for 20 mins with the brake off. Monocytes were isolated by positive selection using anti-CD14 magnetic microbeads using column separation (Miltenyi, Germany) and assessed for percentage CD14-FITC positivity (Miltenyi, Germany) using Amnis Cellstream (Luminex, Texas, United States).

### Dendritic cell culture and stimulation

Human monocyte-derived immature DC (moDC) were seeded at a density of 1 × 10^6^ cells/mL in 6-well plates in 3 mL of RPMI-1640 medium containing 10 % defined low-endotoxin HyClone FBS (Thermo Fischer Scientific, Massachusetts, United States), 1 % penicillin-streptomycin (Lonza, Switzerland), 1 % Fungizone (Sigma Aldrich, California, United States), human granulocyte macrophage colony-stimulating factor (50 ng/mL) (Immunotools, Germany) and human IL-4 (70 ng/mL) (Immunotools, Germany). moDC were incubated in a humidified atmosphere with 5 % CO_2_ at 37 °C. Cells were fed at day 3 with a half medium change and supplemented with fresh cytokines. At day 6, moDCs were assessed for immaturity, with CD11c+ cells exhibiting an immature DC phenotype capable of upregulating maturation and activation markers. Freshly generated moDCs were plated in 96-well plates at 2 × 10^5^ cells in 150 μl RPMI 1640 media supplemented with 10 % defined low-endotoxin HyClone FBS (Fisher Scientific, Massachusetts, United States) and stimulated with irradiated ACM (iACM), or matched background media controls, for 6 h before exposure to 10 μg/mL of ultrapure TLR4 agonist Escherichia coli lipopolysaccharide (LPS-EB; Invivogen, California, United States) overnight for the LPS positive cohort, the LPS negative cohort was not treated with LPS. Supernatants were harvested and stored at −80 °C for further processing, and cells were assessed for expression of surface markers as described below.

### Macrophage culture and stimulation

Human monocyte-derived immature Macrophages (moMɸ) were seeded at a density of 1 × 10^6^ cells/mL in 6-well plates in 3 mL of RPMI-1640 medium containing 10 % FBS (Thermo Fischer Scientific, Massachusetts, United States), 1 % penicillin-streptomycin (Lonza, Switzerland), human macrophage colony-stimulating factor (50 ng/mL) (Immunotools, Germany). moMɸ were incubated in a humidified atmosphere with 5 % CO_2_ at 37 °C. Cells were fed at day 3 with a half medium change supplemented with fresh cytokines. At day 6, moMɸ were assessed for immaturity, with CD68+ cells exhibiting an immature Mɸ phenotype capable of upregulating markers of polarisation following stimulation. Freshly generated moMɸ were plated in 96-well plates at 2 × 10^5^ cells in 150 μl and stimulated with iACM, or matched background media controls, for 6 h before exposure to 100 ng/mL of ultrapure TLR4 agonist E. coli lipopolysaccharide (LPS-EB; Invivogen, California, United States) or 100ng/ml of IL-4 (Immunotools, Germany) overnight for the M0 naïve (untreated) cohort, M1-like LPS stimulated, M2-like IL-4 stimulated. Cells were assessed for expression of surface markers as described below.

### Flow cytometry

All cells were washed with PBS and stained with zombie UV viability dye (Biolegend, California, USA). Antibodies used for DC staining included CD40-FITC, CD11c-Vio-Blue, CD80-PE, CD83-APC-Vio770, CD86-PerCp-Vio700, HLA-DR-Vio-Green, CD54-APC, and PD-L1-PE-Vio770. Antibodies used for Mɸ staining included CD11b-FITC, CD11c-Vio-Blue, CD80-PE, CD206-APC-Vio770, CD86-PerCp-Vio700, HLA-DR-Vio-Green, TIM-3-APC, CD68-PE-Vio770, and CD163-PEVio615 (Miltenyi, Germany). Cells were washed with FACs, fixed, washed, and then resuspended in FACs buffer on 96 well plates. Samples were acquired on Cell stream flow cytometer (Amnis, Luminex, Texas, USA) with compensation performed with positive and negative compensation beads (Miltenyi, Germany, BD Biosciences, UK) with Live/Dead control gated on cells (Biolegend, California, United States). Gating on and analysis of CD11c+ for DC or CD68+ for Mɸ cells was performed using Cell Stream software (Amnis, Luminex, Texas, USA).

### Statistical analysis

All statistics were conducted using GraphPad Prism 9.5 (GraphPad Software, California, United States). A significance level of *p* < 0.05 was used in all analysis and all p-values reported were two-tailed. Friedman testing with Dunn's post hoc correction, was employed for non-parametric testing between paired cohorts. Details of specific statistical tests are given in each corresponding figure legend.

## Results

### Chemotherapy diminishes oxidative phosphorylation in adipose tissue explants which is by oleic acid

Chemotherapy and chemoradiotherapy are known to detrimentally effect adipose tissue function. Whilst obesity, an extreme modulator of adipose tissue function, has been implicated as potentiating influence on the efficacy of these treatments. Agilent seahorse technology was used to assess the influence of chemotherapy or chemoradiotherapy on the metabolic profiles and phenotypes of adipose tissue. Real time metabolic readouts were obtained for OCR, an indicator of oxidative phosphorylation associated metabolism and ECAR, a measurement associated with glycolytic metabolism. OCR:ECAR ratio values were utilised to identify adipose metabolic preferences following treatment.

**Fig. 1:**
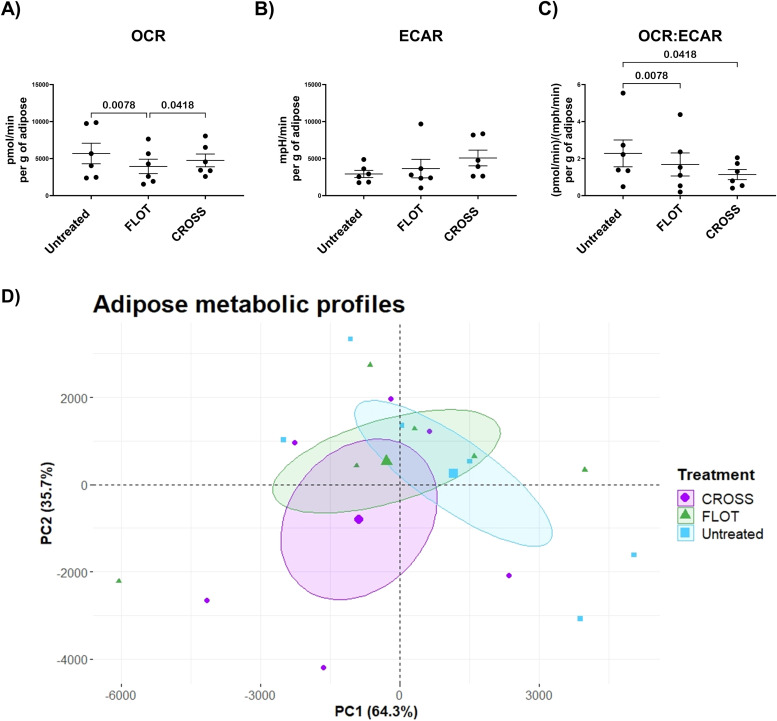
**Chemotherapy diminishes oxidative phosphorylation in adipose tissue explants, whilst both therapies increase glycolytic preferences in adipose explants.** A-C) OCR, ECAR and OCR:ECAR ratio profiles of untreated, chemotherapy treated (FLOT) and chemoradiotherapy treated (CROSS) adipose explants (Friedman test with Dunn's correction). D) PCA plot detailing adipose metabolic profiles through dimensionality reduction. All data expressed as mean ± SEM, *n* = 6, associated *p-value* displayed above comparison, only signifcant results shown.

Here, we observed that the FLOT chemotherapy regimen significantly decreased OCR metabolism in adipose explants compared with untreated and CROSS chemoradiotherapy regimen treated adipose explants. Additionally, both FLOT and CROSS regimens significantly decreased adipose explants reliance on oxidative phosphorylation over glycolysis (Fig. 1**A and C**). Principal component analysis (PCA) of the adipose explant metabolism showed modest separation by treatment groups (Fig. 1**D)**.

### Chemoradiotherapy increases the secreted levels of pro-inflammatory mediators in the adipose secretome

Chemotherapy and Chemoradiotherapy have been established to have widespread effects on the tumour microenvironment through the alteration of a series of pro-inflammatory, immune-inhibitory, and angiogenic mediators both in circulation and at tissue level. To establish a baseline to examine the effects of chemotherapy and chemoradiotherapy on the adipose secretome of cancer patients, a 54-analyte multiplex was used to assess inflammatory mediators comparing against a matched, untreated adipose secretome.

**Fig. 2:**
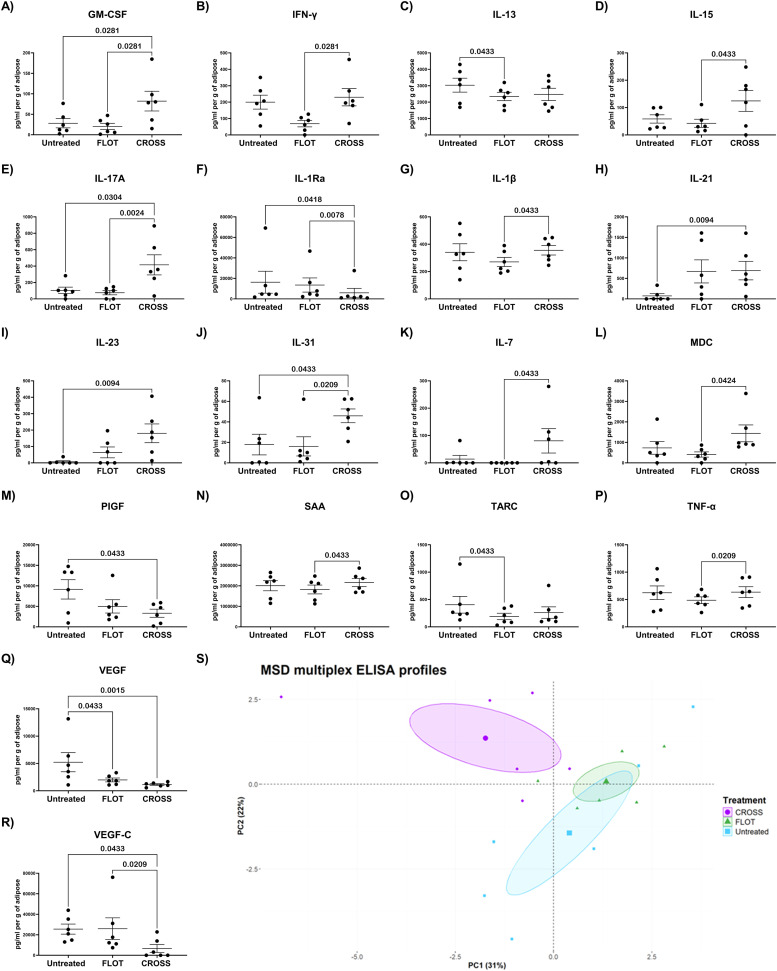
Chemoradiotherapy increases the secreted levels of mediators of immune cell recruitment and decreases secretion of angiogenic mediators in the adipose secretome. A-R) Secreted levels of GM-CSF, IFN-γ, IL-1β, IL-1Ra, IL-7, IL-13, IL-16, IL-17A, IL-21 IL-23, IL-31, MDC, PIGF, SAA, TARC, TNF-α, VEGF, and VEGF-C of untreated, chemotherapy treated (FLOT) and chemoradiotherapy treated (CROSS) adipose explants, (Friedman test with Dunn's correction). S) PCA plot detailing MSD multiplex ELISA analysis of the treated adipose secretome through dimensionality reduction. All data expressed as mean ± SEM, *n* = 6, associated *p-value* displayed above comparison, only signifcant results shown.

FLOT regimen significantly decreased adipose secreted levels of anti-inflammatory, immune-inhibitory and angiogenic mediators compared with untreated adipose, including IL-13, TARC, and VEGF (Fig. 2**C, O and Q**). CROSS regimen significantly decreased adipose secreted levels of a series of anti-inflammatory and angiogenic mediators compared with untreated adipose, including IL-1Rα, PIGF, VEGF, and VEGF-C (Fig. 2**F, M, Q and R)** CROSS regimen further showed diminished adipose secreted levels of anti-inflammatory and angiogenic associated mediators compared with FLOT treated adipose tissue, including IL-1Ra and VEGF-C (Fig. 2**F and R**). CROSS regimen significantly increased adipose secreted levels of a series of pro-inflammatory and immune activating mediators compared with untreated adipose, including GM-CSF, IL-17A, IL-21, IL-23, and IL-31 (Fig. 2**A, E, H, I and J**). Interestingly, CROSS regimen showed significantly elevated adipose secreted levels of a series of pro-inflammatory, immune-activating and vascular injury associated mediators compared with FLOT-treated adipose tissue, including GM-CSF, IFN-γ, IL-1β, IL-7, IL-16, IL-17A, IL-31, MDC, SAA, TNF-α (Fig. 2**A, G, K, D, E, J, L, N and P)**. PCA analysis of the adipose secretome by MSD multiplex ELISA showed little separation by treatment groups (Fig. 2**S)**.

### The chemotherapy treated adipose secretome increases reliance on glycolysis and augments dysfunctional mitochondrial response to stress in oesophageal cancer cells

Cancer cells have been reported to enhance glycolytic metabolism to survive the cytotoxic effects of chemotherapy [31]. Whilst chemoradiotherapy has been shown to decrease cancer metabolism directly following irradiation with a subsequent enhanced glycolysis [32]. To assess the influence of chemotherapy or chemoradiotherapy on the adipose secretome and how this alters cancer cell metabolic profiles MitoStress test and seahorse technology was used. Crystal violet staining was utilised to assess the viability of these cells following culture with the chemotherapy and chemoradiotherapy treated adipose microenvironments after 24 h. As well as acting as a metric for the normalization of these metabolic read-outs.

**Fig. 3:**
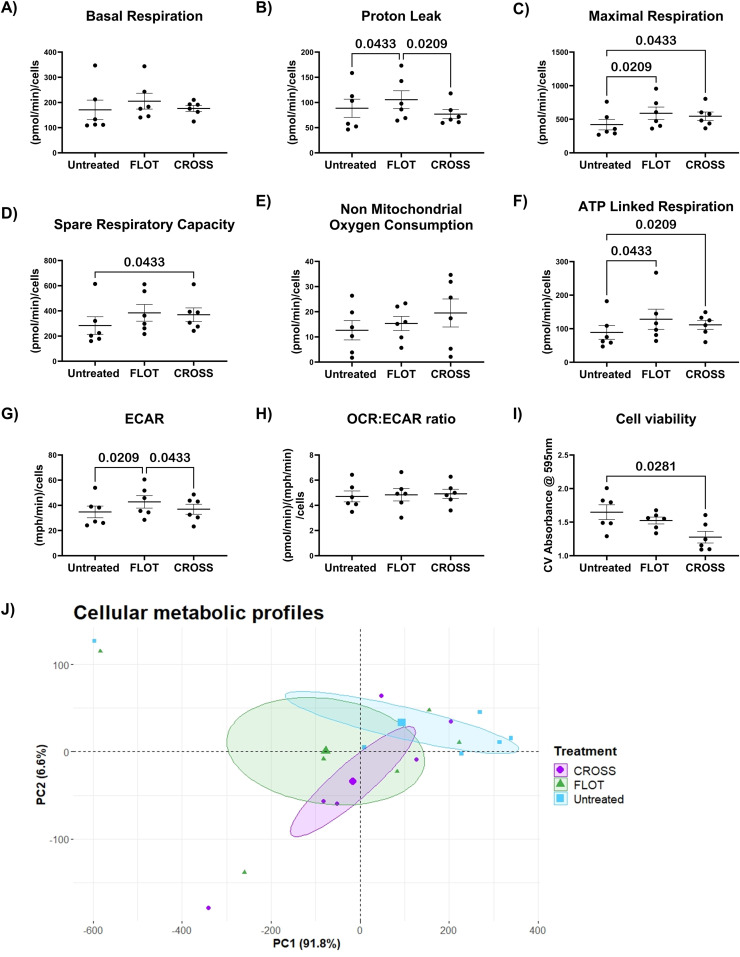
**The chemotherapy treated adipose secretome increases reliance on glycolysis and augments dysfunctional mitochondrial response to stress in oesophageal cancer cells.** A-H) Mitochondrial parameters including basal respiration, proton leak, maximal respiration, spare respiratory capacity, non-mitochondrial oxygen consumption, ATP linked respiration, ECAR and OCR:ECAR ratio of OE33 cell lines following exposure to untreated, chemotherapy treated (FLOT) and chemoradiotherapy treated (CROSS) adipose conditioned media. I) Cell viability following culture with treated adipose conditioned media, (Friedman test with Dunn's correction). J) PCA analysis of OE33 metabolic profiles following culture with the treated adipose secretome through dimensionality reduction. All data expressed as mean ± SEM, *n* = 6, associated *p-value* displayed above comparison, only signifcant results shown.

OE33 oesophageal cancer cells cultured with FLOT-treated adipose secretome demonstrated metabolic profiles associated with mitochondrial dysfunction, including elevated glycolytic metabolism, increased maximal respiration, and increased proton leak (Fig. 3**G, C and B**). OE33 cells cultured with CROSS treated adipose secretome showed increased ATP linked respiration, maximal respiration, and spare respiratory capacity, an indication that cells are still capable of utilising mitochondrial reserves (Fig. 3**F, C and D**). Oesophageal cancer cells cultured with CROSS-treated adipose secretome showed significantly decreased viability following 24-hour incubation, however no significant effects were observed in cells cultured with FLOT-treated adipose secretome (Fig. 3**I**). PCA analysis of OE33 s cultured with the treated adipose secretome showed little separation by treatment groups (Fig. 3**J).**

### Chemotherapy increases DCs maturation markers, whilst all treatments increased immunoinhibitory ligand TIM-3

Chemotherapy and chemoradiotherapy have been reported to enhance DCs antigen presentation [23]. To assess the influence of chemotherapy or chemoradiotherapy on the adipose secretome and how this alters DCs maturation, stimulated DCs were profiled via flow cytometry for a panel of phenotypic and maturation markers following exposure to treated ACM.

**Fig. 4:**
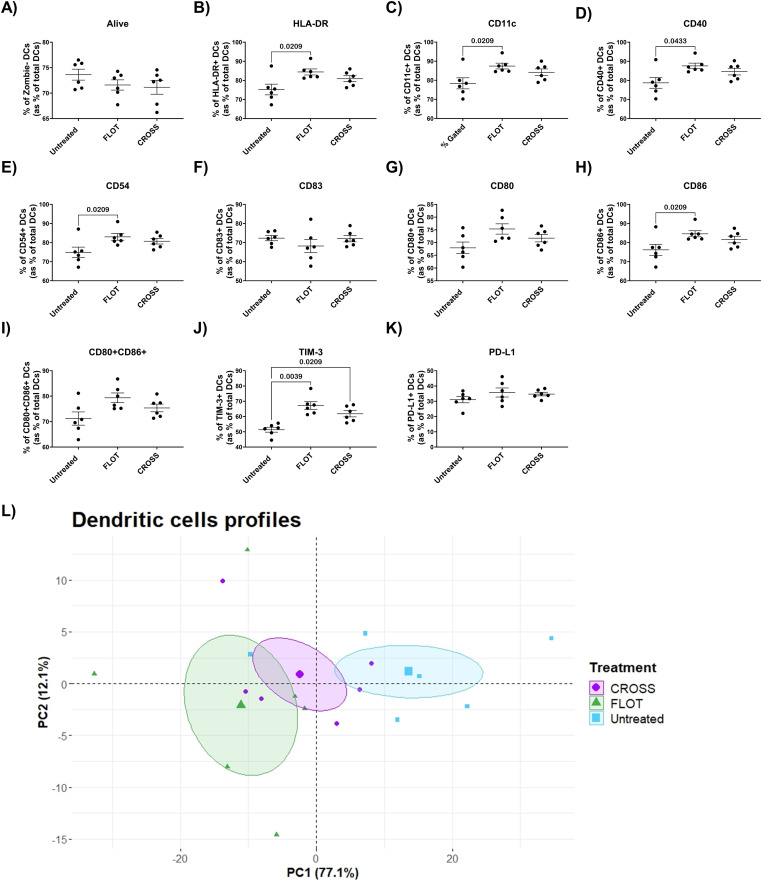
**Chemotherapy increases DCs expression of phenotypic and adhesion associated markers.** A-K) Expression of DC phenotypic and maturation markers including viability, HLA-DR, CD11c CD40, CD54, CD83, CD80, CD86, CD80+CD86+, TIM-3 and PD-L1 following exposure to untreated, chemotherapy-treated (FLOT) and chemoradiotherapy-treated (CROSS) adipose conditioned media, (Friedman test with Dunn's correction). L) PCA analysis of DCs maturation marker expression following culture with the treated adipose secretome through dimensionality reduction. All data expressed as mean ± SEM, *n* = 6, associated *p-value* displayed above comparison, only signifcant results shown.

DCs cultured with the FLOT-treated adipose secretome showed significantly increased expression of a series of phenotypic and maturation ligands, including HLA-DR, CD11c, CD40, CD54, and CD86 (Fig. 4**B, C, D, E and H**). In addition to this, DCs cultured with both FLOT-, and CROSS-treated ACM showed increased expression of immunoinhibitory ligand TIM-3 (Fig. 4**J**). PCA analysis of the DCs cultured with the treated adipose secretome showed increased separation by treatment groups (Fig. 4**L)**.

### Chemotherapy ACM significantly increases anti-inflammatory markers on unpolarised Mɸ whilst chemoradiotherapy significantly increases pro-inflammatory markers

Chemotherapy and chemoradiotherapy have been reported to have pleiotropic effects on Mɸ polarisation. To assess the influence of chemotherapy or chemoradiotherapy on the adipose secretome and how this alters Mɸ maturation, unpolarised Mɸ were assessed via flow cytometry for a panel of phenotypic, pro-inflammatory and anti-inflammatory markers following exposure to these treated ACM.

**Fig. 5:**
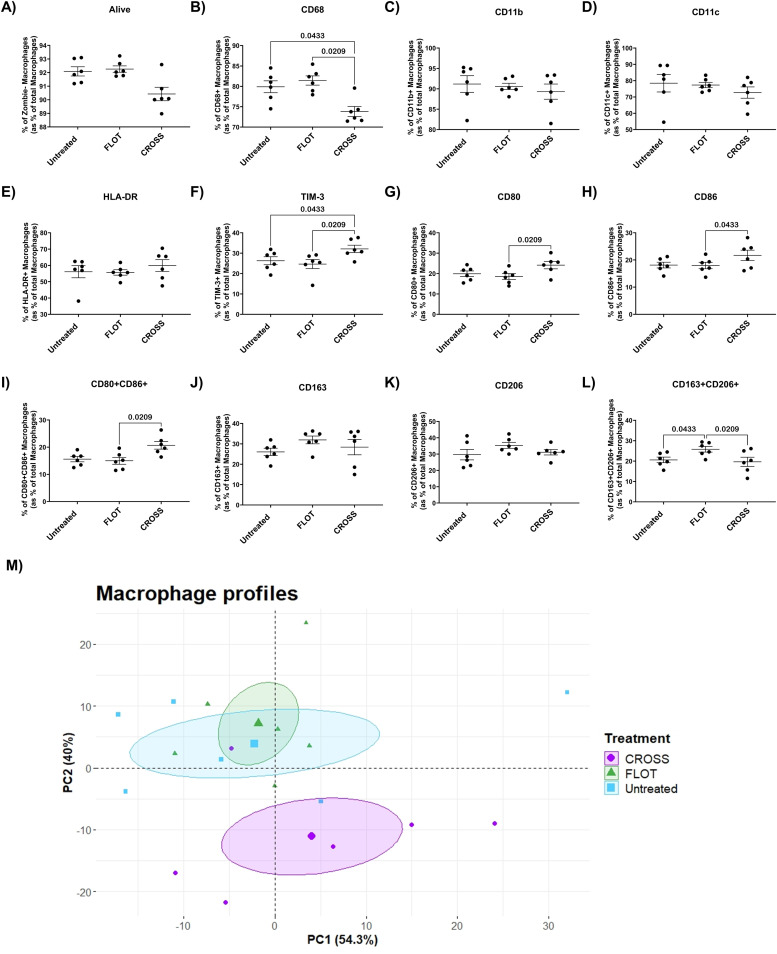
**Chemoradiotherapy treated adipose secretome significantly increases immunoinhibitory markers TIM-3 on unpolarised Mɸ.** A-L) Expression of unpolarised Mɸ phenotypic and immunoinhibitory markers including viability, CD68, CD11b, CD11c, HLA-DR, TIM-3, CD80, CD86, CD80+CD86+, CD163, CD206, and CD163+CD206+ following exposure to untreated, chemotherapy treated (FLOT) and chemoradiotherapy treated (CROSS) adipose conditioned media, (Friedman test with Dunn's correction). L) PCA analysis of Mɸs polarisation marker expression following culture with the treated adipose secretome through dimensionality reduction. All data expressed as mean ± SEM, *n* = 6, associated *p-value* displayed above comparison, only signifcant results shown.

Unpolarised Mɸ cultured with CROSS treated ACM showed significantly decreased expression of phenotypic markers CD68, as well as increased expression of pro-inflammatory associated markers CD80, CD86, CD80^+^CD86^+^, and immune-inhibitory marker TIM-3 (Fig. 5**B, F, G, H and I**). Unpolarised Mɸ cultured with FLOT-treated ACM showed significantly increased co-expression of anti-inflammatory associated markers CD163^+^CD206^+^ (Fig. 5**L**). PCA analysis of Mɸs cultured with treated adipose secretome showed increased separation the CROSS-treated adipose secretome and compared with untreated and FLOT-treated groups (Fig. 5**M**).

### The chemotherapy-treated adipose secretome increases M1 polarised Mɸ expression of pro-inflammatory and immune-inhibitory ligands whilst chemoradiotherapy-treated ACM augments M2 polarised Mɸ expression of anti-inflammatory associated marker CD206

Chemoradiotherapy has been reported to evoke radiation induced inflammation, a response perpetuated by M1-like macrophages [29]. Radiotherapy has been reported to decrease M2-like macrophages and increase pro-inflammatory responses, whilst chemotherapy has been reported to enhance M2-like recruitment [25,29]. To assess the influence of chemotherapy or chemoradiotherapy on the adipose secretome and how this alters Mɸ maturation, M1-polarised Mɸ by LPS and M2-polarised Mɸ by IL-4 were assessed via flow cytometry for a panel of phenotypic, and anti-inflammatory markers following exposure to these treated ACM.

**Fig. 6:**
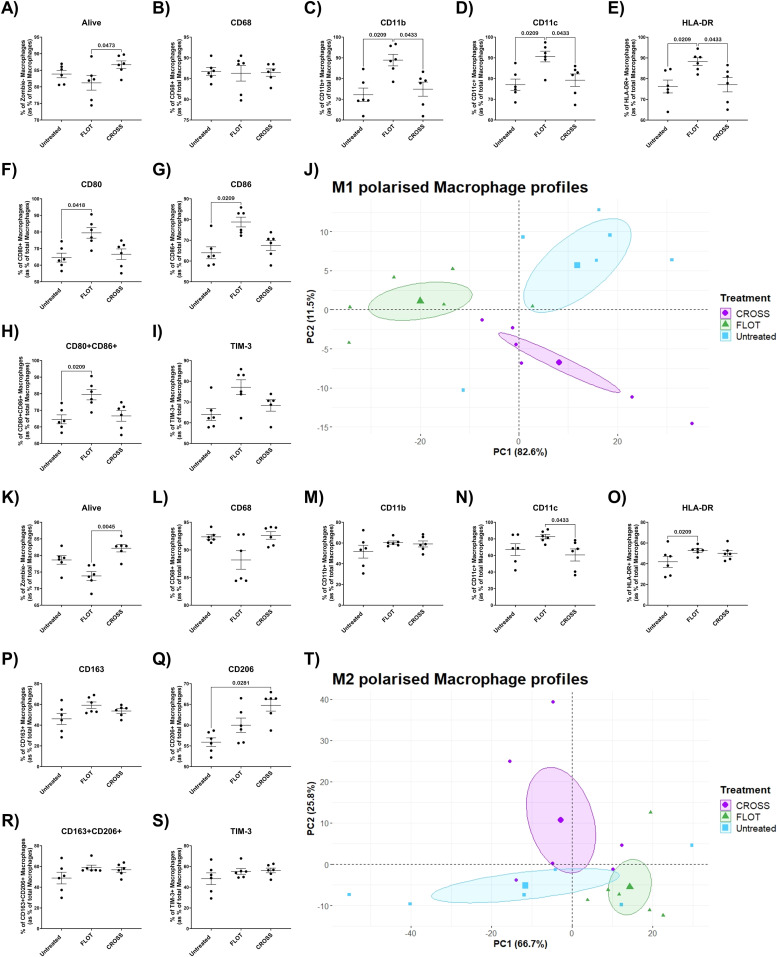
**Chemotherapy treated adipose secretome significantly increases M1 polarised Mɸ expression of phenotypic and immune-inhibitory ligands whilst the chemoradiotherapy treated adipose secretome increases M2 polarised Mɸ expression of CD206.** A-I) Expression of LPS M1 primed Mɸ phenotypic and immunoinhibitory markers including viability, CD68, CD11b, CD11c, HLA-DR, TIM-3, CD80, CD86 as well as co-expression of CD80 and CD86 following exposure to untreated, chemotherapy treated (FLOT) and chemoradiotherapy treated (CROSS) adipose conditioned media, (Friedman test with Dunn's correction). J) PCA analysis of M1-primed Mɸ polarisation marker expression following culture with the treated adipose secretome through dimensionality reduction. K-S) Expression of IL-4 M2 primed Mɸ phenotypic and immunoinhibitory markers including viability, CD68, CD11b, CD11c, HLA-DR, TIM-3, CD163, CD206 as well as co-expression of CD163 and CD206 following exposure to chemotherapy treated (FLOT) and chemoradiotherapy treated (CROSS) adipose conditioned media, (Friedman test with Dunn's correction). T) PCA analysis of M2-primed Mɸ polarisation marker expression following culture with the treated adipose secretome through dimensionality reduction. All data expressed as mean ± SEM, *n* = 6, associated *p-value* displayed above comparison, only signifcant results shown.

M1-polarised Mɸ cultured with FLOT-treated ACM also showed significantly decreased viability compared with Mɸ cultured with CROSS-treated ACM (Fig. 6**A**). M1-polarised Mɸ cultured with FLOT-treated ACM showed significantly increased expression of a series of Mɸ phenotypic and immune-inhibitory associated markers including CD11b, CD11c, HLA-DR, and TIM-3 compared with control ACM (Fig. 6**C, D, E and F**). M1-polarised Mɸ cultured with FLOT-treated ACM showed significantly increased expression of a pro-inflammatory associated Mɸ markers including CD80, CD86, and co-expression of CD80^+^CD86^+^ compared with control ACM (Fig. 6**G, H and I**). PCA analysis of the M1-primed Mɸs cultured with the treated adipose secretome showed increased separation between treatment groups (Fig. 6**J**).

M2-polarised Mɸ cultured with FLOT-treated ACM also showed significantly decreased viability compared with Mɸ cultured with CROSS treated ACM (Fig. 6**K**). M2-polarised Mɸ cultured with FLOT treated ACM also showed significantly increased expression of a series of Mɸ phenotypic markers including CD11c, and HLA-DR compared with control and CROSS-treated ACM (Fig. 6**N and O**). However, M2-polarised Mɸ cultured with CROSS treated ACM showed significantly increased expression of anti-inflammatory associated marker CD206 compared with matched control (Fig. 6**Q**). PCA analysis of the M1-primed Mɸs cultured with the treated adipose secretome showed high separation between treatment groups (Fig. 6 **T**).

## Discussion

The role of adipose tissue and the influence of obesity on cancer treatment response has been contentiously reported. For the first time, this study examined the influence chemotherapy and chemoradiotherapy regimens have on adipose tissue metabolism and its secretome. This study looked to further explore what implications this treated adipose secretome has for cancer cell metabolism, DC maturation and Mɸ polarisation.

In this study, FLOT chemotherapy regimen decreased OCR metabolism and OCR:ECAR ratio in adipose explants compared with untreated adipose. OCR, a measurement of oxidative phosphorylation, has been reported to be unregulated in more aggressive cancers, and inhibition of oxidative phosphorylation has been suggested to enhance the efficacy of chemotherapy [33]. However, a reduction in reactive oxygen species (ROS) production in the presence of prolonged chemotherapy has been shown to be a mitigating factor in its efficacy [34]. Additionally, chemoradiotherapy was observed to decrease OCR:ECAR ratio, indicating a switch in preference to glycolysis. Radiation therapy has been shown to evoke a switch to glycolysis in cancer cells to survive the subsequent induced oxidative stress [35]. These diminished OCR levels in adipose tissue following chemotherapy and elevated reliance on glycolysis following chemoradiotherapy may have more dynamic effects in the wider tumour microenvironment. This switch in metabolic preference could lead to an altered nutrient composition in the adipose tissue which may aid in the development of metastatic niches. Differential metabolism of metabolites such as glutamine, that we have previously seen decreased in obese adipose tissue [36], or fatty acids could lead to an altered adipose milieu that may have opposing effects on cancer cell proliferation and migration. These differential metabolic reactions in adipose tissue in response to chemotherapy and chemoradiotherapy may be adaptions to survive the induction of ROS caused by these therapies. However, the damage induced as a by-product of these cytotoxic therapies may lead to detrimental changes in the adipose tissue biology and secreted factors that could aid the progression of cancer and diminish anti-tumour immunity. Further research is required to elucidate if these aberrant adipose metabolic changes following treatment exposure could be targeted for therapeutic gain.

The adipose secretome has also been reported to influence the tumour microenvironment, as well as mitigating cancer treatment response [4,8]. This study reports adipose tissue exposed to chemoradiotherapy showed increased secreted levels of a series of pro-inflammatory, vascular injury, immune recruiting and activating mediators GM-CSF, IFN-γ, IL-7, IL-16, IL-17A, IL-1β, IL-21, IL-23, IL-31, MDC, SAA, and TNF-α. Previous research has shown increased leukocyte infiltration and myeloid cell activation following radiation therapy, [37]. GM-CSF and MDC are two key cytokines associated with immune cell recruitment and activation. Interestingly, GM-CSF is given in combination with radiotherapy to combat neutropenia, it has also been shown to not increase tumour cell migration [38]. Increases in secreted levels of GM-CSF from adipose tissue following chemoradiotherapy could act beneficially but further research is required to interrogate this link. Additionally, as would be expected following chemoradiotherapy, adipose tissue showed significant increases in secreted levels of pro-inflammatory cytokines including IFN-γ, IL-7, IL-16, IL-17A, IL-1β, IL-21, IL-23, IL-31, and TNF-α. Radiation induced inflammation is a known phenomenon [39], which is central to promoting anti-tumour immunity and regression [40]. Such increases in the secretome of adipose tissue following chemoradiotherapy could enhance the circulatory pro-inflammatory response and aid in tumour cell death. Perhaps unsurprisingly, increased secreted levels of SAA were also observed following chemoradiotherapy, a factor that has previously be linked to radiation-induced damage [41]. Decreased levels of SAA in circulation have previously been associated with diminished overall survival in patients receiving radiotherapy [42]. This increase in the adipose secretome may be influential in identifying a wider pro-inflammatory response following radiotherapy, which could be utilised diagnostically.

Adipose tissue exposed to chemoradiotherapy showed decreased secreted levels of anti-inflammatory and angiogenic mediators IL-1Ra, PIGF, SAA, VEGF, VEGF-C. IL-1Ra has been reported to competitively inhibit pro-inflammatory responses from IL-1 family associated cytokines [43]. This decreased secretion by adipose tissue following exposure to chemoradiotherapy may aid in perpetuating radiation induced inflammation. PIGF, VEGF, and VEGF-C are known promotors of endothelial cell proliferation and [[44], [45], [46]] previous research in rectal cancer biopsies has shown diminished endothelial cell proliferation after radiotherapy [37]. Interestingly, these factors were decreased following chemoradiotherapy, asking whether this response could ameliorate circulating expression of these angiogenic mediators in the wider tumour microenvironment. Of note, adipose tissue exposed to chemotherapy shown decreased secreted levels of immunoinhibitory mediators IL-13 and TARC. IL-13 plays a central role activating a Th2 response and inducing M2-like Mɸ polarisation, both critical factors in diminishing the efficacy of anti-tumour immunity [47]. Similarly, TARC has been reported to play a central role in stimulating Th2 driven immunity [48]. The influence of chemotherapy on the adipose secretome provokes some interesting questions. Here we observe that chemotherapy significantly decreases adipose secreted drivers of Th2 immunity, which are known to support pro-tumour immunity. This leads to the question whether this influence is adipose specific and how this could alter the inflammatory landscape of adipose tissue itself. These findings could specifically prove beneficial in obese OAC patients who suffer from a low grade pro-inflammatory state, who show dysregulated immune responses to typical drivers of pro-inflammatory responses [49].

Cellular metabolism is a multi-faceted mitochondrial process and mitochondrial dysfunction, commonly induced by cancer cells to aid tumour progression, is an even more complex system [14,50]. This study reports increased maximal and ATP-linked respiration by OAC cell line OE33 cultured within the secretome of adipose explants exposed to chemotherapy and chemoradiotherapy. Increased maximal respiration is an indication of response to external stress, whilst increased ATP-linked respiration indicates an increase in ATP demand, which can be linked to cellular proliferation [51]. Additionally, studies have shown that high ATP levels enhance cancer cells resistance to chemotherapy [52]. Interestingly OE33 exposed to chemotherapy-treated ACM showed increased basal respiration and ECAR, whilst cells cultured with chemoradiotherapy showed increased spare respiratory capacity. A high basal OCR indicates that cells are using a higher percentage of the maximal rate of respiration and indicates they may not have enough reserved capacity to meet the cellular energy demands required through oxidative phosphorylation alone [51]. Spare respiratory capacity is depleted under extreme oxidative stress. In the instance where oxidative phosphorylation fails to reach the threshold required to sustain the energetic needs of the cell, glycolysis is stimulated to meet energy demands [53]. This high basal OCR and switch to glycolysis in OE33 cultured with chemotherapy-treated ACM may indicate a higher presence of oxidative stress and mitochondrial dysfunction. In addition to this, OE33 cultured with chemotherapy-treated ACM showed increased levels of proton leak. Proton leak has been linked to a series of factors such as damaged induced to the inner mitochondrial membrane or increased uncoupling protein activity [51]. Uncoupling proteins are known to interfere with ATP production by inducing proton leak, UCP2 in particular has been reported to be expressed in aggressive cancers and has been implicated in promoting chemoresistance [54]. Conclusively, chemotherapy-treated ACM promotes mitochondrial dysfunction and increased reliance on glycolysis in OE33 a metabolic switch which may aid in chemoresistance. These effects are not observed in cells cultured with chemoradiotherapy treated ACM. These findings indicate that the metabolic flexibility evoked by cancer cells to evade cell death and enhance chemoresistance could be further bolstered by the adipose secretome. Such a mechanism could aid cancer cells in the seeding of metastatic niches to survive the cytotoxic effects of chemotherapy indicating this therapy option may not be as beneficial in obese OAC patients.

DC possess a critical role in initiating anti-tumour immunity through antigen presentation. Chemotherapy has previously been reported to increase DC antigen presentation [23], however induction of DC response following radiotherapy was shown to be dependent on an active immune infiltrate already being present [24]. DCs cultured with ACM treated with chemotherapy showed increased expression of HLA-DR, CD11c, CD40, CD54, CD86. CD40 plays an essential role in activating DC maturation leading to increased expression of CD80/CD86 and production of

IL-12 [55], whilst CD54 supports DC adhesion and activation of T cells [56,57]. CD86 is widely reported for its role in DC maturation, polarisation of Th1 response and activation of NK cells [58]. This upregulation of a series of factors that promote DC maturation, and T cell priming may indicate that the chemotherapy treated adipose secretome could aid in the promotion of a wider anti-tumour immune response which may aid in treatment efficacy. While chemotherapies have previously been shown to elicit anti-tumour immune responses [59], identifying that the adipose secretome does not appear to mitigate this phenomenon in DC's cross presenting responses is an interesting finding. However, DCs cultured with ACM treated with chemotherapy or chemoradiotherapy also showed increased expression of TIM-3. TIM-3 expression on DCs has been reported to inhibit antitumour immunity by inhibiting inflammasome activation [60]. This may act as a diminishing influence on the cytotoxicity of these treatments but also highlight a potential therapeutic target. Antagonism of TIM-3 in combination with chemotherapy is currently under investigation [61], inhibition of this immune checkpoint may prove beneficial in OAC potentially aiding poor responses to chemotherapy which are currently observed in this disease setting.

Macrophages in both their pro-inflammatory and anti-inflammatory phenotypes play a central role in cancer progression and treatment resistance. Anti-inflammatory or TAMs have been reported to be induced by chemotherapy [25] as well as mediating chemoresistance [26]. On the other hand, pro-inflammatory macrophages have been reported to play a vital role in radiation induced inflammation, inducing an anti-tumour immunity through the generation of an inflammatory response [29]. This study reports in unpolarised Mɸ, the adipose secretome of explants treated with chemoradiotherapy significantly increased expression of inflammatory markers CD80, CD86 and co-expression of CD80 and CD86. In contrast, Mɸ cultured with chemotherapy-treated ACM showed increased co-expression of CD163 and CD206 markers which are known to be associated with a TAM-like phenotype. It is interesting to observe chemoradiotherapy treated ACM enhancing M1 associated markers and chemotherapy treated ACM upregulating expression of M2 associated markers which speaks to the opposing mechanisms of action these cytotoxic therapies induce. However, it is of note that Mɸ cultured with chemotherapy and chemoradiotherapy treated ACM showed increased expression of TIM-3. TIM-3 has previously been reported to evoke the development of TAMs [62] and expression of TIM-3 on TAMs has been correlated with more aggressive cancers and poorer survival rates in patients [63]. Highlighting again the therapeutic potential of targeting expression of this immune checkpoint in this disease setting.

Interestingly in M1-polarised Mɸ cultured with chemotherapy-treated ACM showed increased expression of CD11b, CD11c, HLA-DR, CD80, CD86, CD80+CD86+ and TIM-3. The chemotherapy treated adipose secretome may perpetuate the effects of Mɸ priming towards a pro-inflammatory phenotype. Such an occurrence could prove as an attractive therapeutic target to diminish the induction of TAMs by chemotherapy regimens [25] but further research is required to investigate if there is any utility in this relationship. M2-polarised Mɸ cultured with chemoradiotherapy showed increased expression of anti-inflammatory associated marker CD206 compared with matched control. CD206 is a powerful inducer of anti-inflammatory response that stimulates the release of cytokines known to resolve inflammation such as IL-10, IL-13, and TGF-β [64]. Mɸ primed by IL-4 for anti-inflammatory response may upregulate expression of CD206 following exposure to ACM treated with chemoradiotherapy to resolve the resulting radiation induced inflammatory signals.

Conclusively for the first time, this study has shown that chemotherapy and chemoradiotherapy differentially alter adipose tissue metabolism and secreted factors, with chemoradiotherapy significantly increasing pro-inflammatory associated mediators. However, the chemotherapy treated-adipose secretome enhanced mitochondrial dysfunction in cancer cells as well as increasing their reliance on glycolysis. Whilst chemotherapy-treated ACM significantly increased DC maturation markers, a comparable increase was observed in M2-like phenotypes in unpolarised Mɸ, which could lead to immunosuppressive effects in the wider tumour microenvironment. Chemoradiotherapy significantly increased M1-like phenotypes in unpolarised Mɸ, this pro-inflammatory state could play a role in enhancing anti-tumour immunity. Adipose tissue is a regulatory organ with so many poorly understood downstream effects but is central to the development of so many obesity-associated cancers. We have demonstrated that chemotherapy and chemoradiotherapy can significantly alter the metabolic preferences and secretome of adipose tissue. Alterations in the adipose secretome could potentiate anti-tumour immunity and treatment efficacy, therefore further interrogation is required to fully elucidate the influence adipose tissue may have in treatment response.

## Funding

This research was funded by Breakthrough Cancer Research, grant number 209740/16104. This research used core equipment funded by the CROSS-cancer research charity.

## CRediT authorship contribution statement

**Fiona O'Connell:** Writing – review & editing, Writing – original draft, Visualization, Methodology, Investigation, Funding acquisition, Formal analysis, Data curation, Conceptualization. **Eimear Mylod:** Writing – review & editing, Resources, Methodology, Data curation. **Noel E. Donlon:** Writing – review & editing, Data curation. **Maria Davern:** Writing – review & editing, Resources, Methodology. **Christine Butler:** Writing – review & editing, Data curation. **Niamh O'Connor:** Writing – review & editing, Data curation. **Meghana S. Menon:** Writing – review & editing, Data curation. **Claire L. Donohoe:** Writing – review & editing, Data curation. **Narayanasamy Ravi:** Writing – review & editing, Data curation. **Derek G. Doherty:** Writing – review & editing, Methodology. **Margaret R. Dunne:** Writing – review & editing, Methodology. **John V. Reynolds:** Writing – review & editing, Data curation. **Helen M. Roche:** Writing – review & editing, Supervision. **Jacintha O'Sullivan:** Writing – review & editing, Writing – original draft, Visualization, Supervision, Resources, Project administration, Methodology, Funding acquisition, Formal analysis, Conceptualization.

## Declaration of competing interest

The authors declare that they have no known competing financial interests or personal relationships that could have appeared to influence the work reported in this paper.

## Appendix Supplementary materials

Image, application 1
