# Supplementary material for: Adipose tissue from oesophageal adenocarcinoma patients is differentially affected by chemotherapy and chemoradiotherapy regimens altering immune cell phenotype and cancer cell metabolism
Source: Transl Oncol. 2025 Feb 17;53:102302. doi: 10.1016/j.tranon.2025.102302 (PMC11876773; doi:10.1016/j.tranon.2025.102302)
Supplement: Supplementary file 1 [file mmc1.docx]

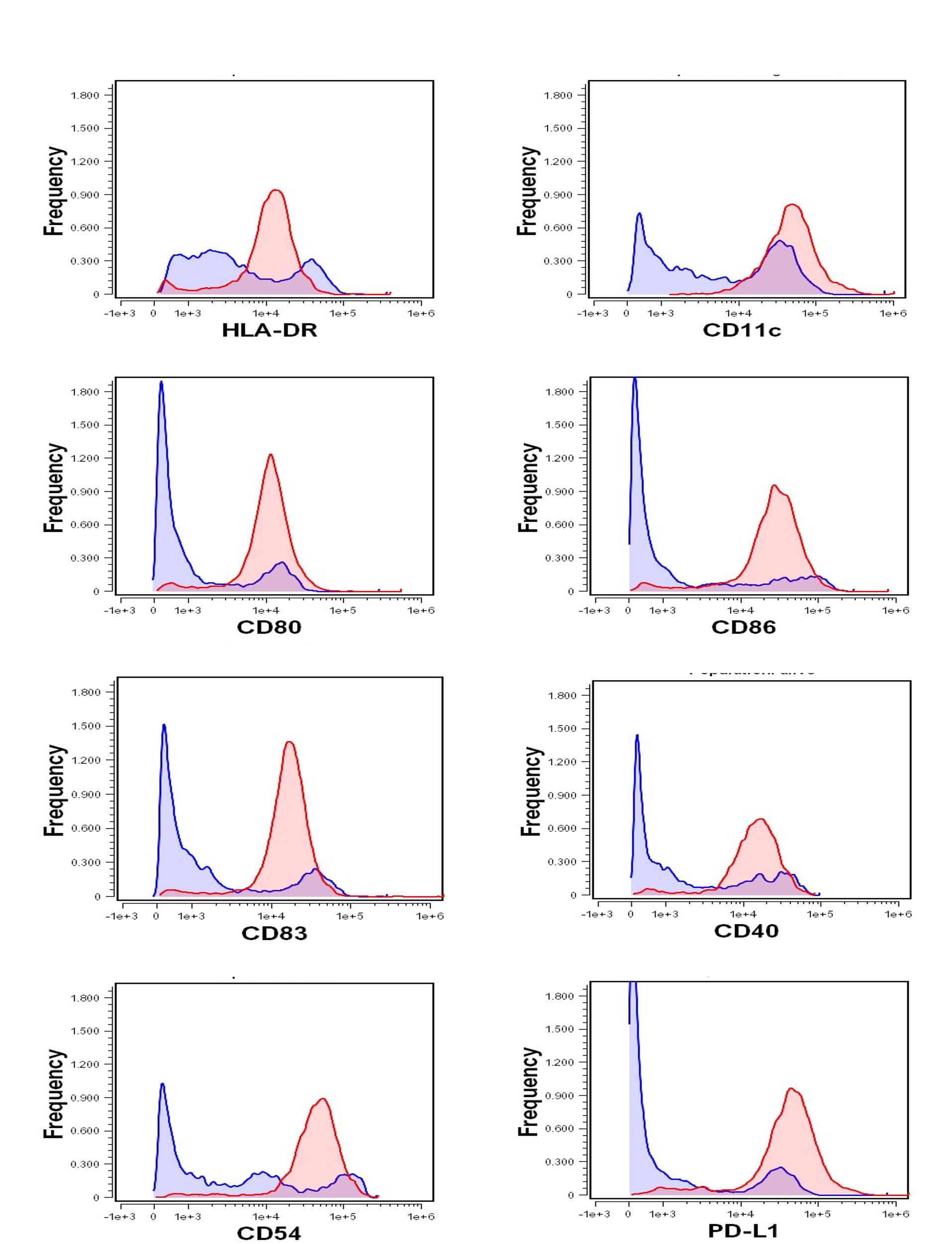
**Supplemental figure 1. Histograms of DC maturation markers following LPS stimulation**

Histograms displaying DC markers HLA-DR, CD11c, CD80, CD86 CD83, CD40, CD54 and PD-L1 which depict DCs response to LPS stimulation. (Blue peaks indicate unstimulated DCs and red peak indicate DCs following exposure to LPS stimulation)
